# PCN-222 metal–organic framework: a selective and highly efficient sorbent for the extraction of aspartame from gum, juice, and diet soft drink before its spectrophotometric determination

**DOI:** 10.1186/s13065-020-00674-6

**Published:** 2020-03-20

**Authors:** Zahra Safaei Moghaddam, Massoud Kaykhaii, Mostafa Khajeh, Ali Reza Oveisi

**Affiliations:** 1 Department of Chemistry, Faculty of Sciences, University of Sistan and Baluchestan, Zahedan, 98135-674 Iran; 2 Department of Chemistry, Faculty of Science, University of Zabol, Zabol, Iran

**Keywords:** Aspartame, Zirconium-based metal–organic framework, PCN-222(Fe), Solid-phase extraction, Diet soft drink analysis, Spectrophotometry

## Abstract

In this paper, we describe synthesis and application of an iron porphyrinc metal–organic framework PCN-222(Fe) for solid phase extraction of aspartame, an artificial non-saccharine sweetener, from gum, juice and diet soft drink samples prior to its determination by spectrophotometry. The mesoporous MOF was synthesized solvo-thermally and characterized by Fourier transform-infrared spectroscopy, powder X-ray diffraction, scanning electron microscopy, and Brunauer–Emmett–Teller techniques. To obtain the best extraction efficiency of aspartame, significant affecting parameters such as pH of sample solution, amount of the sorbent, type and volume of eluting solvent, and adsorption and desorption times were investigated and optimized. Under optimum conditions, the calibration graph for aspartame was linear in the range of 0.1 to 100.0 mg.L^−1^ and relative standard deviation of aspartame was 1.7% (n = 7). Limit of detection of method calculated as 0.019 mg.L^−1^ and the enrichment factor of 350 folds was obtained. Adsorption capacity of synthesized sorbent was found to be 356 mg.g^−1^. Hierarchical porosity, the eight terminal–OH groups of the Zr_6_ node, and hydrogen bonding possibly play vital role for selective adsorption of aspartame. The optimized method was successfully applied to the determination of aspartame in real samples with reasonable recoveries (> 98%).
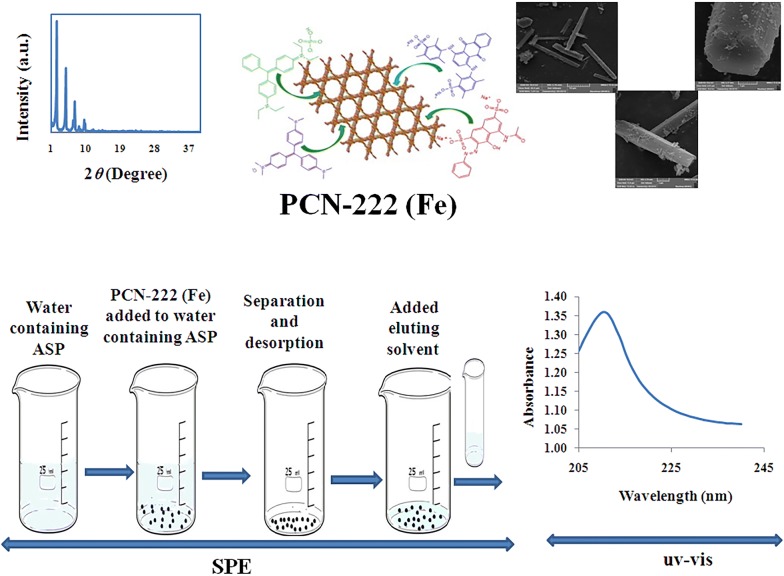

## Introduction

Aspartame (ASP; N-L-a-aspartyl-l-phenylalanine-1-methyl ester), an artificial sweetener, is mostly used in foods, soft drinks, dietary products and preserved fruits for increase product quality and shelf life [1]. There are some witnesses that support there is a relationship between ASP intake and harmful health issues such as obesity, dental caries, carcinogenicity, neurological problems risk of brain tumour rates, and leukaemia [2, 3]. Therefore, it is necessary to develop a fast, simple and sensitive analytical method for detecting ASP in food stuff. According to the joint Food and Agriculture Organization, World Health Organization, and Expert Committee on Food Additives, acceptable daily intake value of ASP is between 0 and 40 mg/kg body mass per day [4].

Spectroscopy (spectrophotometry, colorimetry and chemiluminescence) [5–7], electrochemical techniques [8], and chromatography [9] are the most important analytical methodologies which have been developed for the determination of ASP. Among them, high performance liquid chromatography (HPLC) is the most common technique applied for determination of sweeteners, including ASP; but this method suffers from highly toxic organic solvents, long analysis time and high cost.

Spectrophotometry is an attractive common technique with advantages including high precision, high accuracy, and low cost of analysis [10] which is suitable for determination of many organic and inorganic compounds. The main drawbacks associated with this technique are lack of selectivity and unfeasibility of detecting low concentrations of analytes [11]. These problems can be overcome by applying a proper extraction technique prior to performing spectrophotometry.

Solid phase extraction (SPE) is one of the most important sample preparation methods which has been extensively applied for this purpose to separate and preconcentrate food additives and artificial sweeteners in a wide variety of sample matrices [12]. The advantages of this method include high selectivity, high recovery, good reproducibility, amenable to automation, and low organic solvents requirement [13, 14]. In SPE, sorbent is the most important part which directly affect accuracy, selectivity and sensitivity of the extraction and many researches are focused on the improvement of SPE sorbents [15].

Metal-organic frameworks (MOFs) are a class of crystalline porous materials which are composed of metal ions and organic linkers. They have broad application potentials including adsorption, separation, sensing, drug delivery, detection, catalysis, polymerization, gas storage magnetism, luminescence and removal of toxic materials [16–21] due to their high porosity, large surface areas and tuneable pore size, and in the most cases, the high stability in water [22]. PCN-222(Fe) (PCN stands for porous coordination network) is a mesoporous iron-porhpyrinic zirconium-based MOF with molecular formula of Zr_6_(*μ*_3_-O)_4_(*μ*_3_-OH)_4_(OH)_4_(H_2_O)_4_(FeTCPPCl)_2_, FeTCPPCl = 5,10,15,20-tetrakis (4-carboxyphenyl) porphyrin-iron(III) chloride. The parent-MOF node involves an octahedral Zr_6_ cluster, capped by four *μ*_3_-oxoand four*μ*_3_-OH ligands. Eight of the twelve octahedral edges are linked to FeTCCPCl (as an heme-like ligand) linkers, while the residual of Zr(IV) coordination sites (after activation process with HCl in DMF) are occupied by eight terminal-OH/-H_2_O ligands (non-bridging groups). The accessible eight terminal–OH/–H_2_O group scan be displaced by carboxylate-functionalized molecules via dative bonds [23–26]. This pathway is known as solvent-assisted ligand incorporation method for functionalization of 8-connected Zr_6_ nodes [23–26]. Moreover, the 3D structure of hierarchically porous PCN-222(Fe) typically affords large accessible surface area and high densities of reactant-accessible. Notably, PCN-222(Fe) MOF exhibited extraordinary thermal, mechanical, and chemical stability (stable solution at pH range of 3–10) [27, 28].

Therefore, we decided to prepare, characterize and apply PCN-222(Fe) MOF as a sorbent for SPE of aspartame, a carboxylic acid-containing functional group molecule, from samples with various matrices. Experimental factors affecting extraction were studied and optimized.

## Experimental

### Chemicals

All reagents were of analytical grade and utilized without further purification. Zirconium(IV) chloride (ZrCl_4_), methyl 4-formylbenzoate (C_9_H_8_O_3_), pyrrole (C_4_H_5_N), benzoic acid (C_7_H_6_O_2_), Iron(II) chloride tetrahydrate (FeCl_2_·4H_2_O), propionic acid (C_3_H_8_O_2_) Chloroform (CHCl_3_), ethanol (C_2_H_6_O), methanol (CH_4_O), glucose, sucrose, fructose, sodium ascorbate, cyclamate, hydrochloric acid (HCl), *N,Nʹ*-dimethyl formamide (DMF), sodium hydroxide (NaOH) and tetrahydrofuran (THF) were obtained from Sigma-Aldrich Chemical Company (MO, USA). Reagent grade aspartame was obtained from Merck KGaA (Darmstadt, Germany). Milli-Q^®^ (Merck-Millipore, MA, USA) water (18.3 MΩ cm^−1^) was used throughout all experiments. A stock standard solution of aspartame (1000 mg L^−1^) was prepared by dissolving 1.0000 g of it in 1000 mL of distilled water. Working standard solutions were prepared by serial dilutions of the stock solution prior to analysis.

### Instrumentation

An Agilent (USA) 1200 series HPLC equipped with an Agilent XDB-C18 column (250 mm × 4.6 mm) and an UV detector at fixed wavelength of 210 nm was used for chromatographic separations. Mobile phase was consisted of a mixture of 50 mL of methanol and 50 mL of l % (v/v) triethylammonium acetate buffer (pH 4.5) at the flow rate of 1.0 mL min^−1^. Before analysis, all samples were passed through a 0.22 μm nylon filter. Absorption measurements were carried out with a UV–Vis spectrophotometer (UV-2100 RAYLeigh, Beijing, China) by monitoring the absorbance at maximum wavelength of 210 nm. All experiments were performed at least in triplicates and the mean values were used for optimization. A Metrohm (Switzerland) model 630 pH meter was used for pH measurements. Powder X-ray diffraction (PXRD) patterns were recorded on a Philips X’pert diffractometer (Netherlands) with monochromated Cu Kα radiation (λ = 1.5418 Å) at a range of 1° < 2*θ *< 40°. Fourier transformed infrared (FTIR) spectra were recorded in the range of 4000–5000 cm^−1^ using KBr pellets on a Perkin Elmer Spectrum-FTIR, version 10.01.00 (USA). The morphology and chemical composition of the sample was characterized using scanning electron microscopy (SEM, MIRA3 TESCAN, Czech Republic). The specific surface areas in Brunauer–Emmett–Teller (BET) were determined by N_2_ adsorption–desorption isotherm at liquid nitrogen temperature (TriStar 3020 II, Micromeritics Instrument Corp., Norcross, GA, USA).

### Synthesis of PCN-222(Fe) MOF

PCN-222(Fe) MOF was synthesized through five-step synthesis from commercially available based on a previously reported procedure through five-step synthesis [26, 27].

### Solid phase extraction procedure

A batch SPE method was performed for the extraction of ASP using PCN-222(Fe) MOF as sorbent. 250.0 mL of sample solution was transferred to a beaker and its pH was adjusted to 6.0 using by drop wise addition of either NaOH 0.1 M or HCl 0.1 M solution. 7 mg adsorbent was added to solution and was shaken on a shaker (200 rmp, 10 min) then centrifuged at 4000 rpm for 8 min. The aqueous phase was completely discarded. 700 µL of Ethanol-HCl (99:1 v/v) solution was added to the solid and shook again on a shaker (200 rmp, 15 min). Finally, PCN-222(Fe) MOF was separated from the solution by centrifuging at 4000 rpm for 8 min and the concentration of ASP in elution was determined by UV–Vis spectrophotometry against a blank prepared with the same procedure.

## Results and discussion

### Characterizations of PCN-222(Fe) MOF

**Fig. 1:**
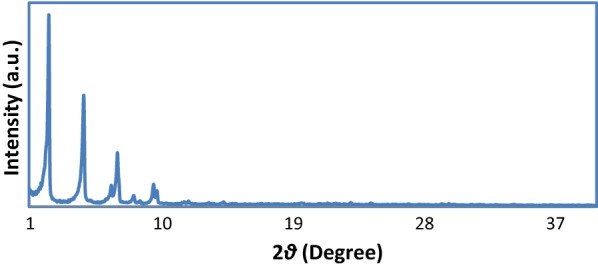
PXRD pattern of synthesized PCN-222(Fe) MOF

The powder X-ray diffraction (PXRD) pattern of the prepared PCN-222(Fe) MOF is shown in Fig. 1. It can be observed that the pattern is similar to previous reports [26, 27]. The intensive peaks at 2*θ *= 2.5, 4.9, 6.6, 7.1 and 9.9º are related to the reflections (1 0 0), (2 0 0), (2 -1 1), (2 0 1), (4 0 0), and (4 -2 1), respectively (CCDC No. 893,545) [22, 27].

**Fig. 2:**
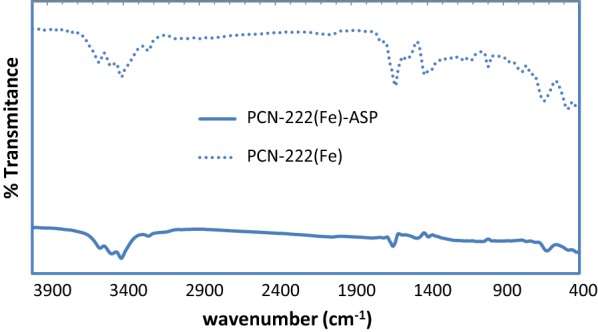
FT-IR spectrum of PCN-222(Fe) and PCN-222(Fe)-ASP

Fourier transform infrared (FTIR) spectrum of PCN-222(Fe) MOF is shown in Fig. 2. The peaks at around 1691 and 1417 cm^−1^ are attributed to strong stretching vibration of -COO (asymmetric) and -COO (symmetric) bonds of the carboxylate groups. The peaks at about 1570, 650 and 712 cm^−1^ are due to the out of plane bending of the C-Hs of phenyl rings [29]. After ASP sorption, the intensity peaks at 1700 cm^−1^ and 1570 cm^−1^ decreased and leading to a shift towards lower wave numbers.

**Fig. 3:**
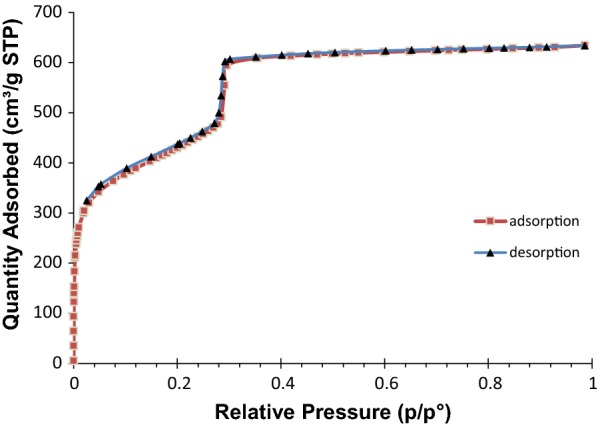
Nitrogen adsorption–desorption isotherms for PCN-222(Fe) at 77 K

**Fig. 4:**
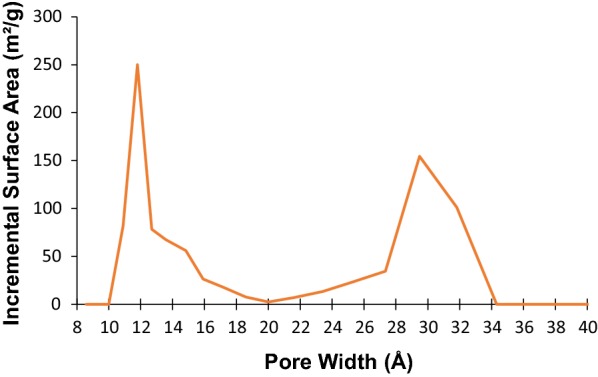
DFT pore size distribution for the PCN-222(Fe)

The porosity of the prepared MOF was measured by nitrogen adsorption–desorption experiments at 77 K. The typical type IV isotherm and a Brunauer–Emmett–Teller (BET) surface area of 1650 m^2^ g^−1^ were obtained, when the activation procedure was applied (Fig. 3). Density functional theory simulation of the N_2_ sorption revealed that the nominal MOF have two types of pores, with sizes of ~ 1.2 nm and ~ 3 nm (Fig. 4), respectively, corresponded to triangular micro channels and hexagonal meso channels.

**Fig. 5:**
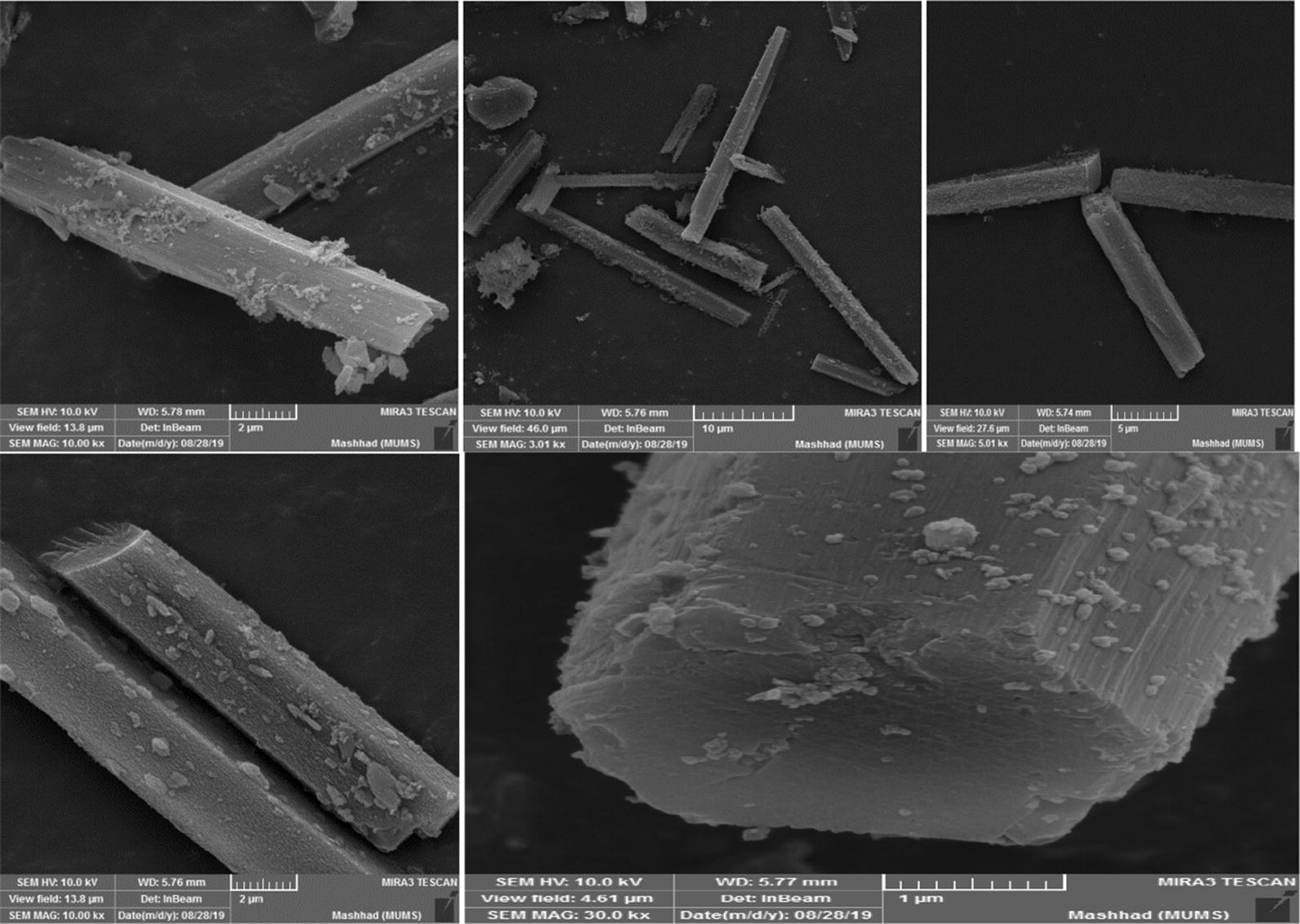
SEM of PCN-222(Fe)

Scanning electron microscope (SEM) image was applied to characterize the morphology of the as-synthesized PCN-222(Fe), and is shown in Fig. 5. As can be seen in the image of metal–organic framework synthesized, that it has typical rod-like structure which was similar to the reported studies [30].

### Optimization of SPE procedure

Several important parameters influencing the extraction efficiency, including pH of the sample solution, amount of adsorbent, type and volume of eluting solvent, and adsorption and desorption time were studied and optimized, as discussed below. A standard solution of 10 mg L^−1^ of ASP was used for this purpose.

#### Effect of pH

**Fig. 6:**
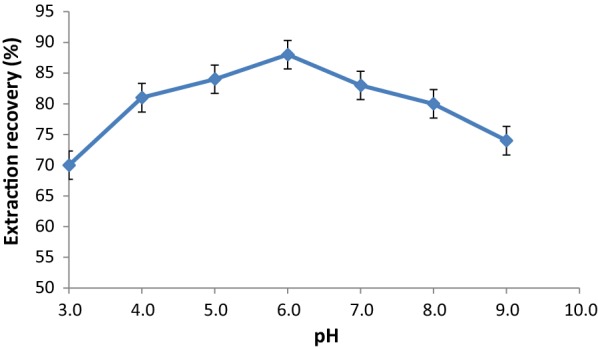
Effect of pH on extraction recovery of ASP (adsorption conditions: 100 µL of 10.0 mg L^−1^ ASP solution; 10 mg of adsorbent; 15 min contact time)

The pH of the sample solution is one of the most critical parameters in the adsorption of ASP on the MOF which shows its influence by two factors: the form of the analyte and surface binding sites on the adsorbent [31]. pH of a series of ASP standard solutions was varied in the range of 3.0–9.0 and results are shown in Fig. 6. As can be seen, the optimum point occurs at pH 6.0. Mechanism of aspartame adsorption on PCN-222(Fe) MOF can be explained with electrostatic interaction between aspartame and the adsorbent. According to a previous research, zero charge point of PCN-222(Fe) MOF was observed at pH = 6.4 [32]. At pHs other than this value, the surface charge of MOF is charged. On the other hand, isoelectric point of ASP was determined as 5.25 (pK_1_ 3.18, pK_2_ 7.82) [33]. At pH around 6, MOF is positively charged, while ASP is in anionic form, therefore recovery increases.

#### Effect of type and volume of the eluent

**Fig. 7:**
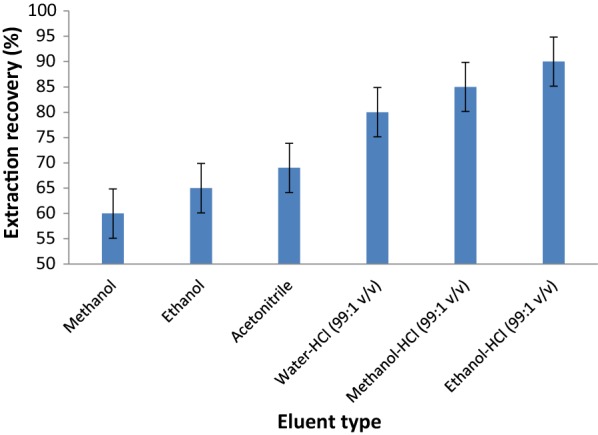
Effect of eluent type on extraction recovery of ASP (adsorption conditions: 100 µL of 10.0 mg L^−1^ ASP solution; 10 mg of adsorbent; 15 min contact time; pH:6)

The effect of eluent type on recovery of ASP from MOF was studied. Methanol, ethanol, acetonitrile, water–HCl (99:1 v/v), methanol-HCl (99:1 v/v) and ethanol-HCl (99:1 v/v) were tested. Acid concentration should be kept at the lowest possible level to prevent MOF degradation. The obtained results are shown in Fig. 7. A quantitative recovery for the ASP was obtained using ethanol-HCl (99:1 v/v) as eluent, maybe because acid can convert ASP from its anionic form to neutral.

**Fig. 8:**
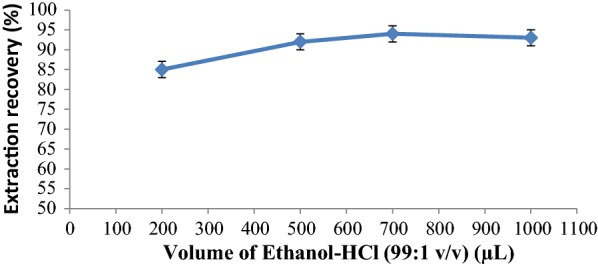
Effect of volume of eluting solvent on extraction recovery of ASP (adsorption conditions: 100 µL of 10.0 mg L^−1^ ASP solution; 10 mg of adsorbent; 15 min contact time; pH:6)

The effect of the volume of eluting solvent was investigated at the range of 200–1000 µL (Fig. 8). The results show that 700 µL of ethanol-HCl (99:1 v/v) is favorable to obtain maximum extraction recovery of ASP. At higher volumes, a diversing effect is observing, probably due to the effect of dilution of eluted ASP.

#### Influence of the amount of sorbent

**Fig. 9:**
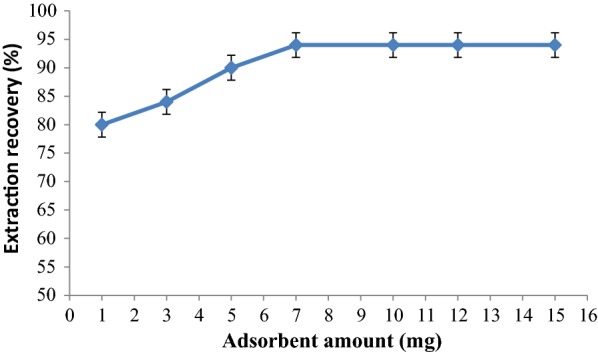
Effect of amount of PCN-222(Fe) MOF on extraction recovery of ASP (adsorption conditions: 100 µL of 10.0 mg L^−1^ ASP solution; 15 min contact time; pH:6)

The effect of dosage of adsorbent on extraction recovery of ASP in the range of 1.0–10.0 mg is shown in Fig. 9. The maximum extraction recovery was obtained when the amount of the MOF was 7.0 mg. As can be seen, only a tiny amount of adsorbent was enough to extract the ASP because of its high adsorption capacity. Thus, 7 mg of the PCN-222(Fe) was utilized for further experiments.

#### Effect of adsorption and elution times

**Fig. 10:**
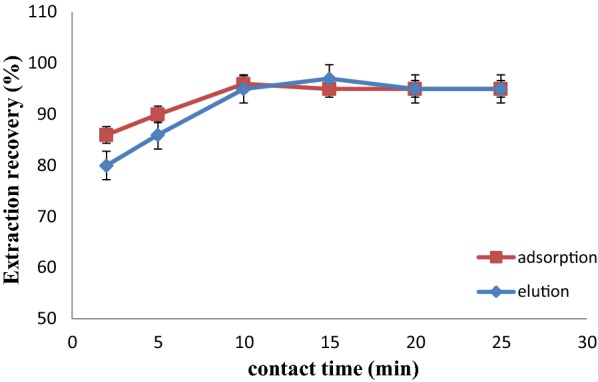
Effect of adsorption and elution contact times on extraction recovery of ASP (adsorption conditions: 100 µL of 10.0 mg L^−1^ ASP solution; pH:6; 7 mg of adsorbent)

To reach the best equilibrium time, it is necessary to optimize contact time for the analyte adsorption and desorption. Contact times of 2, 5, 10, 15, 20 and 25 min was tested for both extraction and elusion. Results are depicted in Fig. 10. The time required achieving equilibrium for adsorption and elution was 10 min and 15 min, respectively. This fast kinetic is due to high specific surface area and large pores of the synthesized MOF.

#### *Effect of* ionic strength

The impact of ionic strength on performance of ASP extraction was studied by making the sample solution 0.0–1.0 mol L^−1^ with respect to sodium chloride, while other experimental conditions were kept constant. The results showed that the absorbance signal of ASP was almost independent of the ionic strength of the solution; hence, all extraction experiments were performed without salt addition.

#### Effect of sample volume

In order to obtain a high preconcentration factor, the influence of the sample volume on the extraction efficiency of ASP was investigated in the range of 10.0–500.0 mL. The results showed that the recovery of ASP was very efficient (> 98%) in a sample volume range of 10–250 mL and after that, recovery decreases. Hence, 250 mL of the sample solution was used in the subsequent studies.

Enrichment factor (EF), defined as the ratio of the sample volume of 250 mL to the final elution volume of 700 µL, was expected to be 357 folds which was closed to the 350 folds experimentally determined.

#### Effect of interferences

The selectivity of the present method was investigated by analyzing standard solutions containing 10 mg L^−1^ of ASP in the presence of high amounts of common compounds which are normally co-exist with ASP. The tolerance limit was defined as the maximum concentration of foreign species causing an error of less than ± 5% in the determination of ASP. The results which are summarized in Table 1 showed that there are no interferences from the tested species on preconcentration and determination of ASP by suggested method.

**Table 1:** Effect of interference of foreign species on the tolerance limits of 10 mg L^−1^ ASP in the aqueous samples under the optimized condition (acceptable error less than ± 5%)

Foreign species	Tolerance limit (mg L^−1^)	Recovery (%)
Ascorbate	500	98
Glucose Fructose, Sucrose	400 200	95 97
Cyclamate	100	98

#### Linear range, limit of detection and precision

Under the optimum conditions, the linear range, detection limit, coefficient of determination, enrichment factor, accuracy and precision of the suggested method were obtained and summarized in Table 2. The calibration curve was linear over a concentration range of 0.1–100 mg L^−1^ with a coefficient of determination (R^2^) of 0.997. Limit od detection (LOD), obtained from 3(S_d_)_blank_/m (where (S_d_)_blank_ is the standard deviation of ten consecutive measurements of the blank and *m* is the slope of calibration curve), was 0.019 mg L^−1^. For evaluation of the sorption capacity of MOF, under the optimum conditions of ASP adsorption, 7 mg of this sorbent was added to 250.0 mL of 10 mg L^−1^ standard solution of ASP and after reaching equilibrium, concentration of remaining ASP was determined using an HPLC by direct injection of 10 µL of the solution and comparison to a calibration curve. Peak areas were used for quantifications.

**Table 2:** Analytical figure of merit for SPE extraction of ASP

Parameter	Analytical feature
Equation of calibration curve	A = 0.0769C_ASP_ + 0.2534
Dynamic range (mg L^−1^)	0. 1–100
R^2^ (determination coefficient)	0.9978
Repeatability (RSD %, n = 7)	1.70
Limit of detection (mg L^−1^)	0.019
Enrichment factor	350
Capacity of sorbent (mg g^−1^)	356

Adsorption capacity was found to be 356 mg g^−1^ which was calculated from the following equation [34]:

$$q_{e} = \frac{{\left( {C_{0} - C_{e} } \right) \times V}}{m}$$

where C_0_ and C_e_ are initial and equilibrium concentrations of ASP, V (L) is volume of sample solution and m (g) is the adsorbent dosage. The relative standard deviation (RSD %) of the seven replicate measurements for the same solution was < 1.7%. A comparison between the figures of merit of the method applied in this work with other absorbents used for preconcentration and analysis of ASP are summarized in Table 3. As can be seen, and despite expensive and sophisticated instruments used in some methods, still the PCN-222(Fe) MOF sorbent has higher sorption capacity and better LOD due to having large pore size.

**Table 3:** Comparison of the proposed method with other methods for the determination of ASP

Sorbent	Detection technique	LOD (mg.L^−1^)	Sorbent capacity (mg.g^−1^)	linear range (mg.L^−1^)	Enhancement factor	RSD (%)	Ref.
Sephadex G-25	Spectrophotometry-FIA	0.3	NM	0.001 –0.2	NM	1	[36]
C_18_cartridges	HPLC/UV	NM	NM	NM	NM	3.1	[37]
Monolithic molecularly imprinted polymer	Capillary electrophoresis	NM	NM	0.0001–0.0004	NM	2.7	[38]
Tetraethylenepentamine functionalized Fe_3_O_4_ magnetic polymer	HPLC	0.14	NM	0.005–0.05	NM	3.8	[39]
Ethylenediamine-functionalized magnetic polymer	Ultra-fast liquid chromatography–mass spectrometry	0.15	NM	0.005–0.5	NM	1.1–2.8	[40]
PCN-222(Fe) MOF	Spectrophotometry	0.019	356	0.0–100	357	1.7	This work

*NM* Not mentioned

### Real sample analysis

To assess the performance of this method for the analysis of real samples in complicated matrices, the proposed procedure was applied to the determination of ASP in three different samples, i.e. a soft cola drink, a peach juice and a bubble gum.

**Fig. 11:**
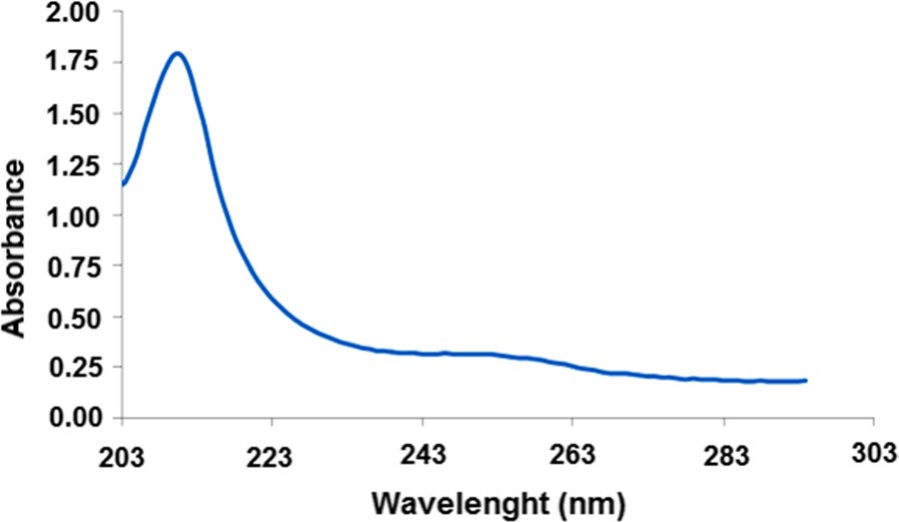
Spectrum of cola drink sample spiked with 50 µg L^−1^ of ASP after MOF-SPE extraction

Juice was degassed and homogenized for 10 min in an ultrasonic bath and cola drink was degassed by putting on a shaker for 15 min. For bubble gum, one stick (weighing 2.7 g) of it was broken to small pieces (approximately 3 × 3 mm) and transferred to a 120 mL volumetric flask and mixture of acetic acid, water and chloroform (1:50:25 v/v) was added to it and stirred at high speed for 10 min. All final solutions were filtered through 0.45-μm filters and finally 10-mL aliquot of each one was diluted in a 100-mL volumetric flask prior to analysis. Detectable amount of ASP was observed in all samples (Table 4). To validate trueness of the analyses, a standard HPLC method [35] was also performed. In order to better evaluate the matrix effect, samples were spiked by adding the appropriate amounts of ASP and analyzed according to the MOF-SPE/spectrophotometric method. Average recoveries ranged from 97% to 104%, with RSDs between 1.0 and 3.6% (n = 3) were obtained which clearly show that this procedure can be successfully applied to trace level determination of the ASP sweetener in various samples. As an example, spectrum of a cola sample spiked with 50 µg L^−1^ of ASP and extracted by MOF-SPE is depicted in Fig. 11.

**Table 4:** Determination of ASP in sweetener samples (n = 3)

Sample	ASP content (µg L^−1^)			Recovery (%)	RSD (%)
	Added	Found by HPLC	Found by SPE-MOF		
Peach juice	0	60	61	101	2.2
	50	111	112	104	2.1
	500	550	549	98	1.9
Bubble gum	0	20	18	90	3.6
	50	70	69	98	2.5
	500	521	519	100	1.0
Soft cola drink	0	100	98	98	1.6
	50	151	150	100	2.5
	500	600	598	99.6	3.6

## Conclusion

In this paper, selective batch-mode solid-phase extraction of aspartame was performed by means of a mesoporous porphyrinic metal–organic framework, PCN-222(Fe), followed by its spectrophotometric determination. The MOF showed high adsorption capacity and high extraction efficiency toward this analyte. Method applicability was demonstrated by analysis of three real samples, including soft cola drink, peach juice and bubble gum and results were compared to standard HPLC analysis with satisfactory results. This method has high preconcentration factor (350), good RSD (< 1.7%) and very low detection limit (19 µg L^−1^). No toxic organic solvents were used durin extraction and elusion. Spectrophotometric instrumentations own merits of simplicity, cheapness, portability and so on.

## Data Availability

All data and analyzed or generated during this investigation are included in the manuscript.

## Abbreviations

ASP: Aspartame
HPLC: High performance liquid chromatography
SPE: Solid-phase extraction
MOF: Metal–organic framework
PCN: Porous coordination network
FTIR: Fourier transformed infrared
SEM: Scanning electron microscopy
PXRD: Powder X-ray diffraction
LOD: The limit of detection
S_d_: Standard deviation
RSD: Relative standard deviation
BET: Brunauer–Emmett–Teller

## References

[1] Fatibello-Filho O, Marcolino-Junior LH, Pereira AV (1999). Solid phase reactor with copper (II) phosphate for flow-injection spectrophotometric determination of aspartame in tabletop sweeteners. Anal Chim Acta.

[2] Schernhammer ES, Bertrand KA, Birmann BM, Sampson L, Willett WC, Feskanich D (2012). Consumption of artificial sweetener—and sugar-containing soda and risk of lymphoma and leukemia in men and women. Am J Clin Nutr.

[3] Olney W, Farber NB, Spitznagel E, Robins LN (1996). Increasing brain tumor rates: is there a link to aspartame. J Neuropathol Exp Neurol.

[4] Summary of Evaluations Performed by the Joint FAO/WHO Expert Committee on Food Additives (JECFA) 1956–1997 (first through forty-ninth meetings), FAO & WHO, 1999

[5] Niu W, Kong H, Wang H, Zhang Y, Zhang S, Zhang XA (2012). Chemiluminescence sensor array for discriminating natural sugars and artificial sweeteners. Anal Bioanal Chem.

[6] Llamas NE, Di Nezio MS, Palomeque ME, Fernández Band BS (2008). Direct determination of saccharin and acesulfame-K in sweeteners and fruit juices powders. Food Anal Methods.

[7] Musto CJ, Lim SH, Suslick KS (2009). Colorimetric detection and identification of natural and artificial sweeteners. Anal Chem.

[8] Pierini GD, Llamas NE, Fragoso WD, Lemos SG, Nezio MS, Centurion ME (2013). Simultaneous determination of acesulfame-K and aspartame using linear sweep voltammetry and multivariate calibration. Microchem J.

[9] Grembecka M, Baran P, Błazewicz A, Fijałek Z, Szefer P (2014). Simultaneous determination of aspartame, acesulfame–K, saccharin, citric acid and sodium benzoate in various food products using HPLC–CAD–UV/DAD. Eur Food Res Technol.

[10] Kaykhaii M, Ghasemi E (2013). Room temperature ionic liquid-based dispersive liquid–liquid microextraction of uranium in water samples before spectrophotometric determination. Anal Methods.

[11] Kaykhaii M, Sargazi M (2014). Comparison of two novel in-syringe dispersive liquid-liquid microextraction techniques for the determination of iodide in water samples using spectrophotometry. Spectrochim Acta A Mol Biomol Spect.

[12] Wasik A, McCourt J, Buchgraber M (2007). Simultaneous determination of ninei ntense sweeteners in foodstuffs by high performance liquid chromatography and evaporative light scattering detection. J Chromatogr A.

[13] Khajeh M, Dastafkan K (2012). Silver nanoparticles attached to silica gel as a new solid phase adsorbent for preconcentration and determination of iron from biological samples. Appl Spectrosc.

[14] Kaykhaii M, Yahyavi H, Hashemi M, Khoshroo MR (2016). A simple graphene-based pipette tip solid-phase extraction of malondialdehyde from human plasma and its determination by spectrofluorometry. Anal Bioanal Chem.

[15] Liu Y, Gao Z, Wu R, Wang Z, Chen X, Chan TWD (2017). Magnetic porous carbon derived from a bimetallic metal–organic framework for magnetic solid-phase extraction of organo chlorine pesticides from drinking and environmental water samples. J Chromatogr A.

[16] Furukawa H, Cordova KE, O’Keeffe M, Yaghi OM (2013). The chemistry and applications of metal-organic frameworks. Science.

[17] Demir S, Usta S, Tamar H, Ulusoy M (2017). Solvent free utilization and selective coupling of epichlorohydrin with carbon dioxide over zirconium metal-organic frameworks. Micropor Mesopor Mat.

[18] Moghaddam ZS, Kaykhaii M, Khajeh M, Oveisi AR (2018). Synthesis of UiO-66-OH zirconium metal-organic framework and its application for selective extraction and trace determination of thorium in water samples by spectrophotometry. Spectrochim Acta A Mol Biomol Spectrosc.

[19] Bobbitt NS, Mendonca ML, Howarth AJ, Islamoglu T, Hupp JT, Farha OK, Snurr RQ (2017). Metal-organic frameworks for the removal of toxic industrial chemicals and chemical warfare agents. Chem Soc Rev.

[20] Adil K, Belmabkhout Y, Pillai RS, Cadiau A, Bhatt PM, Assen AH, Maurin G, Eddaoudi M (2017). Gas/vapour separation using ultra-microporous metal-organic frameworks: insights into the structure/separation relationship. Chem Soc Rev.

[21] Kobielska PA, Howarth AJ, Farha OK, Nayak S (2018). Metal–organic frameworks for heavy metal removal from water. Coord Chem Rev.

[22] Jia X, Zhao P, Ye X, Zhang L, Wang T, Chen Q, Hou X (2016). A novel metalorganic framework composite MIL-101(Cr)@GO as an efficient sorbent in dispersive micro-solid phase extraction coupling with UHPLC-MS/MS for the determination of sulfonamides in milk samples. Talanta.

[23] Deria P, Mondloch JE, Tylianakis E, Ghosh P, Bury W, Snurr RQ, Hupp JT, Farha OK (2013). Perfluoroalkane functionalization of NU-1000 via solvent-assisted ligand incorporation: synthesis and CO_2_ adsorption studies. J Am Chem Soc.

[24] Howarth AJ, Buru CT, Liu Y, Ploskonka AM, Hartlieb KJ, McEntee M, Mahle JJ, Buchanan JH, Durke EM, Al-Juaid SS, Stoddart JF, DeCoste JB, Hupp JT, Farha OK (2017). Postsynthetic incorporation of a singlet oxygen photosensitizer in a metal-organic framework for fast and selective oxidative detoxification of sulfur mustard. Chem Eur J.

[25] Deria P, Bury W, Hupp JT, Farha OK (2014). Versatile functionalization of the NU-1000 platform by solvent-assisted ligand incorporation. Chem Commun.

[26] Ghaleno MR, Moghaddam MG, Khajeh M, Oveisi AR, Bohlooli M (2019). Iron species supported on a mesoporous zirconium metal-organic framework for visible light driven synthesis of quinazolin-4(3*H*)-ones through one-pot three-step tandem reaction. J Colloid interf sci.

[27] Feng D, Gu ZY, Li JR, Jiang HL, Wei Z, Zhou HC (2012). Zirconium metalloporphyrin PCN-222: mesoporous metal–organic frameworks with ultrahigh stability as biomimetic catalysts. Angew Chem Int Ed.

[28] Rezaei Kahkha MR, Daliran S, Oveisi AR, Kaykhaii M, Sepehri Z (2017). The mesoporous porphyrinic zirconium metal-organic framework for pipette-tip solid-phase extraction of mercury from fish samples followed by cold vapor atomic absorption spectrometric determination. Food Anal Methods.

[29] Aghayan M, Mahmoudi A, Sohrabi S, Dehghanpour S, Nazari K, Mohammadian-Tabrizi N (2019). Micellar catalysis of an iron (III)-MOF: enhanced biosensing characteristics. Anal Methods.

[30] Li H, Cao X, Zhang C, Yu Q, Zhao Z, Niu X, Sun X, Liu Y, Ma L, Li Z (2017). Enhanced adsorptive removal of anionic and cationic dyes from single or mixed dye solutions using MOF PCN-222. RSC Adv.

[31] Ma J, Yao Z, Hou L, Lu W, Yang Q, Li J, Chen L (2016). Metal organic frameworks (MOFs) for magnetic solid-phase extraction of pyrazole/pyrrole pesticides in environmental water samples followed by HPLC-DAD determination. Talanta.

[32] Sarker M, Shin S, Jeong JH, Jhung SH (2019). Mesoporous metal-organic framework PCN-222(Fe): promising adsorbent for removal of big anionic and cationic dyes from water. Chem Eng J.

[33] Garcia-Jimenez JF, Valencia MC, Capitan-Vallvey LF (2006). Improved multi-analyte determination of the Intense sweeteners aspartame and acesulfame-k with a solid sensing zone implemented in an FIA scheme. Anal Lett.

[34] Rezaei-Kahkha MR, Kaykhaii M (2016). Removal of uranium(vi) from aqueous solution using modified multiwalled carbon nanotubes: kienetick, isotherm and thermodynamic study. Poll Res.

[35] Issaq HJ, Weiss D, Ridlon C, Fox SD, Muschik GM (1986). The determination of aspartame in diet soft drinks by high performance liquid chromatography. J Liq Chromatogr.

[36] Capitán-Vallvey LF, Valencia MC, Arana Nicolás E, García-Jiménez JF (2006). Resolution of an intense sweetener mixture by use of a flow injection sensor with on-line solid-phase extraction application to saccharin and aspartame in sweets and drinks. Anal Bioanal Chem.

[37] Moors M, Telxena CRRR, Jmndar M, Massart DL (1991). Solid-phase extraction of the preservatives sorbic acid and benzoic acid and the artificial sweeteners aspartame and saccharin. Anal Chim Acta.

[38] An JY, Azizov S, Kumar AP, Lee YI (2018). Quantitative analysis of artificial sweeteners by capillary electrophoresis with a dual-capillary design of molecularly imprinted solid-phase extractor. Bull Korean Chem Soc.

[39] Zhao YG, Cai MQ, Chen XH, Pan SD, Yao SS, Jin MC (2013). Analysis of nine food additives in wine by dispersive solid-phase extraction and reversed-phase high performance liquid chromatography. Food Res Int.

[40] Chena XH, Zhaoa YG, Shenc HY, Jin MC (2012). Application of dispersive solid-phase extraction and ultra-fast liquid chromatography–tandem quadrupole mass spectrometry in food additive residue analysis of red wine. J Chromatogr A.

